# Locally Advanced Non-Small Cell Lung Cancer: Clinical Outcome, Toxicity and Predictive Factors in Patients Treated with Hypofractionated Sequential or Exclusive Radiotherapy

**DOI:** 10.3390/curroncol29070388

**Published:** 2022-07-12

**Authors:** Maria Massaro, Davide Franceschini, Ruggero Spoto, Luca Dominici, Ciro Franzese, Davide Baldaccini, Beatrice Marini, Luciana di Cristina, Marco A. Marzo, Lorenzo lo Faro, Lucia Paganini, Giacomo Reggiori, Carmela Galdieri, Alberto Testori, Marta Scorsetti

**Affiliations:** 1 Radiotherapy and Radiosurgery Department, IRCCS Humanitas Research Hospital, Via Manzoni 56, Rozzano, 20089 Milan, Italy; maria.massaro@cancercenter.humanitas.it (M.M.); ruggero.spoto@cancercenter.humanitas.it (R.S.); luca.dominici@cancercenter.humanitas.it (L.D.); ciro.franzese@hunimed.eu (C.F.); davide.baldaccini@cancercenter.humanitas.it (D.B.); beatrice.marini@cancercenter.humanitas.it (B.M.); luciana.dicristina@cancercenter.humanitas.it (L.d.C.); antonio.marzo@cancercenter.humanitas.it (M.A.M.); lorenzo.lofaro@cancercenter.humanitas.it (L.l.F.); lucia.paganini@cancercenter.humanitas.it (L.P.); giacomo.reggiori@cancercenter.humanitas.it (G.R.); carmela.galdieri@humanitas.it (C.G.); marta.scorsetti@hunimed.eu (M.S.); 2 Department of Biomedical Sciences, Humanitas University, Via Rita Levi Montalcini 4, Pieve Emanuele, 20090 Milan, Italy; 3 Division of Thoracic Surgery, IRCCS Humanitas Research Hospital, Via Manzoni 56, Rozzano, 20089 Milan, Italy; alberto.testori@humanitas.it

**Keywords:** locally advanced non-small cell lung cancer, hypofractionated radiotherapy, Volumetric Modulated Arc Therapy, toxicity, clinical outcome

## Abstract

Background: This study evaluated the outcome, toxicity and predictive factors in patients unfit for concurrent chemo-radiotherapy (CT-RT) treated with hypofractionated sequential CT-RT or exclusive radiotherapy (RT) for locally advanced non-small cell lung cancer (LA-NSCLC). Methods: We included patients affected by LA-NSCLC (stage IIA-IVA) treated with a total dose of 50–60 Gy in 20 fractions. The primary outcomes were local control (LC), distant metastasis-free survival (DMFS), progression-free survival (PFS) and overall survival (OS). Univariate analysis was used to correlate outcomes with prognostic factors. Results: Between 2011 and 2019, 210 patients were treated, 113 (53.8%) with sequential CT-RT and 97 (46.2%) with exclusive RT. After a median follow-up of 15.3 months, 74 patients (35.2%) had a local progression and 133 (63.3%) had a distant progression. The one-, two- and five-year LC were 73.6%, 55.3% and 47.9%, respectively. At the time of analysis, 167 patients (79.5%) died. The one-, two- and five-year OS were 64.7%, 36% and 20%, respectively. PTV volume correlated with PFS (*p* = 0.001) and LC (*p* = 0.005). Acute and late toxicity occurred in 82% and 26% of patients. Conclusions: Albeit with the known limitations of a retrospective and heterogeneous study, our work shows that hypofractionated sequential CT-RT or exclusive RT offer a good local control and toxicity profile and a promising survival rate in LA-NSCLC patients unfit for the concurrent CT-RT scheme.

## 1. Introduction

Lung cancer remains the second most common primary tumor after prostate cancer in males and after breast cancer in females and the first cause of cancer death in both sexes, despite recent improvements [1].

Radiotherapy (RT) is the mainstay local treatment for patients with inoperable or unresectable stage I to III disease [2]. However, while the introduction of Stereotactic Body Radiation Therapy (SBRT) has revolutionized the outcomes of radiotherapy for stage I and selected stage II patients, the local control of conventional radiotherapy for stage II and III NSCLC patients remains largely unsatisfactory, with the 3-year local–regional progression rate up to 62% [3,4].

Recently, the results of the PACIFIC trial have changed the landscape, with the introduction of consolidation immunotherapy (IT) after definitive chemo-radiotherapy [5]. Despite the previously unseen results with a median OS of 47.5 months (the highest ever reported for unresectable LA-NSCLC), there is still room for improvement. Moreover, not all patients are eligible for Durvalumab, due to comorbidities, performance status (PS) or low PDL1 expression.

It is known that higher RT doses are associated with a better tumor control probability [6,7,8]; however, attempts to translate this in clinical results have often failed because of an increased pulmonary and cardiac toxicity. The failure of RTOG 0617 is emblematic in this sense [9].

This unmet need is particularly relevant for patients treated with sequential or exclusive RT. In this clinical scenario, doses up to 80 and 87 Gy have been estimated as necessary to reach the highest possible 2-year OS, without considering the contribution of IT [10]. In the same work, the authors also estimated a possible 5–10% improvement in 2-year OS with hypofractionated dose escalation

On this basis, we conducted a retrospective analysis of patients treated with hypofractionated sequential or exclusive RT in our institution in a pre-PACIFIC era in order to identify the predictive factors of early recurrence and mortality.

## 2. Materials and Methods

### 2.1. Study Population

Patients affected by unresectable NSCLC stage IIA-IVA who were treated with sequential or exclusive hypofractionated RT with 20 fraction schedules in our institution were included in this retrospective analysis. The patients were extracted from internal archives. Inoperability was defined in a multidisciplinary evaluation and could be related to clinical or technical reasons. The patients were excluded from the analysis if they received adjuvant immunotherapy after RT-CT or if they did not receive hypofractionated RT. Demographic, clinical and treatment data were collected from medical charts. The Charlson Comorbidity Index (CCI) was calculated for every patient, and the G8 score was calculated for patients older than 70 years old. The histological confirmation of a primary tumor was available in almost all cases, apart from six cases where the biopsy was contraindicated. Details about chemotherapy, if administered, were also collected.

### 2.2. Treatment Description

All patients underwent radiotherapy; the prescribed dose ranged from 50 to 60 Gy in 20 fractions. All patients underwent simulation 4D-CT with a medium of contrast—in the absence of contraindications—in the supine position and were immobilized with a thermoplastic mask. 18-Fluorodeoxyglucose Positron Emission Tomography (18-FDG PET)/CT was available for the majority of patients and coregistered to simulation CT for delineation purposes. Gross Tumor Volume (GTV) was defined as primary tumor and pathologic lymph nodes (defined according to cytology or histology, when available, or based on radiological suspicion when cytology or histology was not performed). GTV was then modified according to respiratory motion to create the Internal Target Volume (ITV). The ITV was then expanded by 5–8 mm and subsequently modified for anatomical barriers in order to create the Clinical Target Volume (CTV). Another 5 mm isotropic expansion was generated from CTV to obtain the Planning Target Volume (PTV). At least 95% of the prescribed dose was required to cover at least 95% of the PTV. All of the patients were treated with Volumetric Modulated Arc Therapy (VMAT) in its Rapid Arc form. The dose received by the heart, lungs and esophagus was recorded and analyzed.

### 2.3. Follow-Up Schedule

Acute (<3 months from RT) and late toxicity (>3 months from RT) were recorded and scored according to the Common Terminology Criteria for Adverse Events (CTCAE) v.5.0.

Follow-up was performed every 3 months for the first 2 years after radiation therapy and then every 6 months for the other 3 years. Physical examination, performance status, treatment-related adverse effects, blood count, lung function tests (if indicated) and total body CT with the contrast medium were assessed at follow-up. A post-treatment 18 FDG PET/CT was performed if there was suspicion of tumor recurrence or progression at the CT images.

### 2.4. Statistical Analysis

The statistical analysis was performed using Stata 14. The Kaplan Meier analysis was applied to assess local control (LC), distant metastasis-free survival (DMFS), progression-free survival (PFS) and overall survival (OS). Univariate analysis was used to correlate LC, PFS, DMFS and OS to factors potentially predictive of early recurrence and mortality, including patient-related factors (performance status, smoking habit, pulmonary and cardiological comorbidities, Charlson Comorbidity Index, G8 score), tumor-related factors (stage disease, histology, molecular and biomarker analysis in the case of adenocarcinoma, PDL-1 status) and treatment-related factors (chemotherapy and radiotherapy characteristics and schemes). A *p*-value ≤ 0.05 was considered statistically significant.

## 3. Results

Between June 2011 and September 2019, 210 patients were treated with hypofractionated RT. In detail, 113 patients (53.8%) were treated with sequential RT, and the remaining 97 patients (46.2%) were treated with exclusive RT. The demographic and treatment characteristics are summarized in Table 1 and Table 2, respectively. The median age at the time of diagnosis was 72 years old (range 43–93 years).

After a median follow-up of 15.3 months (range 1.9–94.1 months), 74 patients (35.2%) had local progression and 133 patients (63.3%) had distant progression. Twenty-eight patients (13,3%) remained disease-free at the last follow-up.

**Figure 1:**
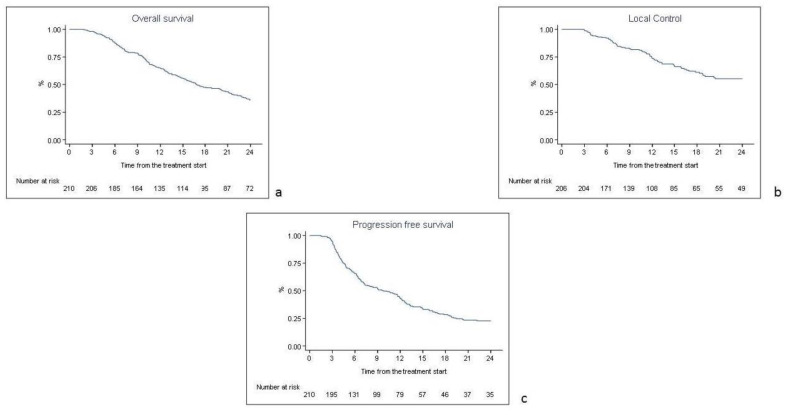
Kaplan Meyer curves for overall survival (**a**), local control (**b**) and progression-free survival (**c**).

At the time of the analysis, 167 patients (79.5%) died. The one-, two- and five-year OS rates were 64.7% (95% CI 57.8–70.7%), 36% (95% CI 29.5–42.6%) and 20% (95% CI 14.2–25.8%), respectively (Figure 1a). The one-, two- and five-year LC rates were 73.6% (95% CI 66.2–79.6%), 55.3% (95% CI 46.5–63.2%) and 47.9% (95% CI 38.1–57%), respectively (Figure 1b). Among the 74 locally progressed patients, 29 patients (39.2%) progressed to the primary tumor, 17 patients (23%) progressed to the locoregional nodes and the other 28 patients (37.8%) had a progression on both the primary and locoregional nodes. The one-, two- and five-year DMFS rates were 50% (95% CI 42.7–56.6%), 33.7% (95% CI 26.7–41%) and 25% (95% CI 17.6–32.7%), respectively. In the analysis of 133 distant progressed patients, the most common progression sites were the: central nervous system (25 patients, 18.8%), pleura/lungs (29 patients, 21.8%), liver (13 patients, 9.7%), bone (12 patients, 9%), metastatic nodes (8 patients, 6%) and adrenal glands (4 patients, 3%), and the other 42 patients (31.7%) had a multi-organ progression. The one-, two- and five-year PFS rates were 43.2% (95% CI 36.2–50%), 22.7% (95% CI 17–29%) and 15% (95% CI 9.8–21.1%), respectively (Figure 1c).

At the univariate analysis, performance status (HR 1.34, 95% CI 1.05–1.71, *p* = 0.01) and cardiological comorbidities (HR 1.43, 95% CI 1.03–1.98, *p* = 0.03) influenced the OS. Albeit with a borderline statistical significance, exclusive RT was correlated with a worse OS (HR 1.32, 95% CI 0.97–1.79, *p* = 0.07). At the univariate analysis for DMFS and PFS, the TNM stage emerged as a negative prognostic factor (HR 1.30, 95% CI 1.13–1.49, *p* = 0.00 for DMFS; HR 1.25, 95% CI 1.10–1.41, *p* = 0.00 for PFS), while adenocarcinoma histology was correlated with a better PFS (HR 0.80, 95% CI 0.66–0.98, *p* = 0.03) and DMFS (HR 0.77, 95% CI 0.61–0.96, *p* = 0.026). Additionally, PTV volume, as a continuous variable, correlated with PFS (HR 1.0, 95% CI 1.00–1.00, *p* = 0.001) and LC (HR 1.00, 95% CI 1.00–1.00, *p* = 0.005).

Among the 113 patients (53.8%) undergoing sequential RT, chemo-radiation treatment was well tolerated: all but one completed the treatment without any interruption. The median follow-up was 16.3 months (range 1–93.7 months), compared with a median follow-up of 14.9 months (range 2–89 months) among the exclusive subset of patients. Comparing the LC and OS rates of the two subgroups of patients, a better outcome of the sequential regime after 2 and 5 years was found. After 2 and 5 years from the treatment, the LC rate for the sequential arm was 62% (95% CI 0.5–0.7) and 54% (95% CI 0.4–0.6), respectively, while the two- and five-year LC rates for the exclusive subgroup were 46.6% (95% CI 33.3–59) and 40% (95% CI 26–53.5), respectively. Regarding the OS rate of the sequential treatment scheme, the 2- and 5-year OS were 41% (95% CI 0.32–0.50) and 27% (95% CI 0.18–0.36), respectively, compared with the 2- and 5-year OS rates of 30% (95% CI 21–39.2) and 11.6% (95% CI 6–20) for the exclusive regimen.

Acute toxicity was recorded in 82% of patients, while late adverse events occurred in 26% of cases. The most common acute toxicities were G1 and G2 and included asthenia (46 patients, 21.8%), dyspnea (25 patients, 11.9%), dysphagia (87 patients, 41.4%) and cough (68 patients, 32.3%); two patients experienced G3 pneumonitis and one patient had G5 pneumonitis. Late esophageal toxicity and dyspnea occurred in 6 (2.8%) and 16 (7.5%) patients, respectively. There were nine cases (4.3%) of G4 pneumonitis, and one patient had G5 pneumonitis. The radiation-associated toxicities are shown in Table 3.

## 4. Discussion

Although the astonishing results of Durvalumab as a consolidation treatment after chemo-radiotherapy increased the therapeutic chances, frailty, PS, comorbidities, autoimmune diseases, PDL1 ≤ 1%, etc. limit the accessibility of such treatment package. Moreover, a non-negligible proportion of patients are not suitable for CT, so RT remains the only curative chance.

Potentiating the efficacy of thoracic RT is still a medical need. In the present work, we report a large retrospective series of LA-NSCLC patients treated with a 20-fraction hypofractionated regimen in an attempt to improve the outcomes.

Indeed, hypofractionation could be a therapeutic option to overcome natural lung cancer radioresistance by reducing the overall treatment time. Moreover, considering the Mehta formula, the reduction in overall treatment time can increase the Biologically Effective Dose (BED) [11] and reduce the accelerated repopulation of tumor cells that could start 4–6 weeks after the beginning of treatment [12].

Alaswad [13] et al. conducted a study to determine the accuracy of a TCP model in describing the clinical outcomes of various fractionation schemes for NSCLC. They analized 2713 patients with early-stage NSCLC treated by three-dimensional conformal radiation therapy (3D-CRT), continuous hyperfractionated accelerated radiotherapy (CHART) or stereotactic ablative radiotherapy (SABR). The authors found that the TCP model used accurately estimated local tumour control one, two and three years post-treatment for all three treatment modalities and that both CHART and SABR were superior to 3D-CRT. Their findings also highlighted the relevance of a short overall treatment time, since, for fractionation schemes completed in fewer than 28 days, the repopulation was negligible.

Several clinical experiences with a variety of radiotherapy fractionations tried to investigate the accelerated RT regimen.

The relationship between BED and OS was supported by the work of Kaster and colleagues. They reported, in a systematic review, the outcome of 22 studies of exclusive hypofractionated RT for LA-NSCLC. The delivered dose ranged from 45 to 85.5 Gy and the median survival ranged from 7.4 months to 21.4 months. The authors found a linear relationship between BED and OS, with a benefit of 0.36–0.7% every 1 Gy increment, and hypofractionated RT, with an overall treatment time of ≤6 weeks, was predicted to be more beneficial than prolonged conventionally fractionated RT [14].

Among the prospective experiences, in 2013, Cannon et al. reported a hypofractionated phase 1 trial of 79 patients. These patients were treated with a mean total dose of 63.25 Gy in 25 fractions (range 57–85.5 Gy) without concurrent CT. With a median follow-up of 17 months (range 2.3–79 months), the 3-year OS was similar to that in our results (29% vs. 25.7%). No G3 pneumonitis was observed; however, with a longer follow-up, G4–5 toxicity occurred in six patients, as was observed in our analysis, recording a G4 late pneumonitis rate of 4.3% (9 patients) and one patient with G5 late pneumonitis. This study identified that late grade 4–5 toxicity was ascribed to damage to the central and perihilar structures and was correlated with a total dose (*p* = 0.004) and with a dose to the proximal bronchial tree. Most of the patients received a total dose of at least 75 Gy. We were not able to find a similar correlation among our patients [15].

In 2020, Kong et al. experimented with a sequential CT-RT schedule delivering, at most, 15 once-daily fractions (total dose range 44–60 Gy) for 42 patients with unresectable stage III NSCLC. After a median follow-up of 46 months (range 41–59 months) the median OS was 47 months. The one-, two- and five-year OS rates were 81%, 69% and 32%, respectively, higher than our one-, two- and five-year OS rates of 64.7%, 36% and 20%, respectively. However, the incidence of G3 lung toxicity was 9.5%, and that of G5 lung toxicity was 4.8%. A total of 12% of the patients experienced acute G3 esophagitis, and 2% had G4 esophageal toxicity [16].

A recent narrative review of the literature conducted by the AIRO-Lung Working Group identified all of the studies from 2007 including patients with LA-NSCLC treated with hypofractionated RT with radical intent, with a minimal dose per fraction of 2.4 Gy and with or without concurrent CT. They found an extreme heterogeneity in terms of RT-treatment schedules. Twenty-nine studies were identified, including 2614 patients. The patients were divided into a concurrent CT-RT group and an RT-alone group. In the RT group (1730 patients), the delivered dose ranged from 45 to 85.5 Gy, with a dose/fraction from 2.4 to 4 Gy. The actuarial 2-year PFS ranged from 13 to 57.8%, slightly lower than the 2-year PFS of 22.7% in our analysis. Comparing the OS, we highlighted a substantially overlapping OS rate; indeed, the authors recorded 1- and 2-year OS ranges of 51.3–95% and 22–68.7%, respectively, versus 64.7% and 36% in our analysis. On the contrary, the late toxicity profile was mildly worse than ours, both for late esophageal toxicity (0–16% vs. 2.8% in our study) and pulmonary toxicity (0–47% vs. 9.9%) [17].

Regarding the comparison between hypofractionated RT and conventional RT, in 2012, Mauguen and colleagues found a strong correlation between survival and overall treatment time in LA-NSCLC. They performed a meta-analysis of 10 trials (2000 patients), in which modified fractionation improved OS as compared with conventional schedules (HR 0.88, 95%CI 0.80–0.97; *p* = 0.009), resulting in an absolute benefit of 2.5% (8.3% to 10.8%) at 5 years. As expected, the use of modified RT increased the risk of acute esophageal toxicity from 9% to 19% (*p* < 0.001) [18]. More recently, another analysis of the National Cancer Database (NCDB) was performed to compare the outcome of exclusive hypofractionated RT and conventional RT in patients with stage III NSCLC. A total of 6490 patients were evaluated: 5378 received conventional RT and 1112 received hypofractionated RT. The median conventional RT dose was 66 Gy in 2 Gy fractions vs 58.5 Gy in 2.5 Gy fractions for hypofractionated RT. On an initial analysis, hypofractionated RT was associated with an inferior OS (median 9.9 vs. 11.1 months, *p* < 0.001), but after adjusting for the imbalance in covariates such as age, BED10, T-stage and N-stage, the difference in survival was no longer significant (*p* = 0.1). Nevertheless, the limits of this study included the absence of information regarding tumor control and cancer-specific survival and the lack of a toxicity profile [19].

All these data confirm that the available literature encloses a wide variety of dose prescriptions and a respective wide range of survivals and toxicities. Our results are in line with the analyzed available literature with an adequate toxicity profile. The large number of patients, the homogeneous prescribed dose (total dose range 50–60 Gy delivered in 20 fractions) and the RT technique employed represent the strengths of this study. At the same time, our study is limited by the retrospective nature of the analysis, the heterogeneity of the disease and the patients’ characteristics and the confounding factor of the different systemic therapies received in the case of sequential CT-RT. It is also difficult to compare our results with the other series of hypofractionated RT in the literature, since the doses/fractionations, the patients’ selection criteria, the use of systemic therapies and many other confounding variables are present.

## 5. Conclusions

Our data show that hypofractionated sequential CT-RT or exclusive RT offer a good local control and an acceptable survival rate in LA-NSCLC patients unfit for the concurrent CT-RT scheme. In this way, frail patients could also benefit from radical treatments and are able to be assured of a good tolerance to therapy. The target volume was related to PFS and LC. The exclusive RT was associated with a worse OS compared to the sequential scheme.

## Figures and Tables

**Table 1:** Patients’ characteristics (*n* = 210).

Patients’ Characteristics	
Age	
Median	72 years
Range	43–93 years
≤70	80 patients (38%)
>70	130 patients (62%)
PS ECOG	
0–1	183 patients (87%)
2–3	27 patients (13%)
Smoke	
Yes	64 patients (30.5%)
No	14 patients (6.7%)
Previous	132 patients (62.8%)
Cardiological comorbidities	
Yes	76 patients (36%)
No	134 patients (64%)
Pulmonary comorbidities	
Yes	152 patients (72%)
No	58 patients (28%)
Charlson Comorbidity Index	
≤6	109 patients (52%)
>6	101 patients (48%)
G8 Score	
≤12	81 patients (38.6%)
>12	129 patients (61.4%)
Histology	
Adenocarcinoma	97 patients (47%)
Squamous	94 patients (44%)
Non-small cells	2 patients (1%)
Other	17 patients (8%)
T-stage	
T0–T2	100 patients (47.6%)
T3–T4	110 patients (52.4%)
N-stage	
N0–N1	67 patients (32%)
N2–N3	143 patients (68%)
TNM (VIII ed.)	
II (A-B)	35 patients (16.6%)
III (A-B-C)	147 patients (70%)
IVA	28 patients (13.4%)
Molecular and Biomarker Analysis (in adenocarcinoma)	
Wild-type	49 patients (46%)
EGFR	5 patients (5%)
ALK	3 patients (2.4%)
ROS-1	0 patients (0%)
Not available	49 patients (46%)
PDL-1	
<1%	8 (4%)
<25%	2 (1%)
25–50%	4 (2%)
>50%	9 (4%)
Not available	185 (89%)

PS ECOG: Performance Status according to the Eastern Cooperative Oncology Group; EGFR: Epidermal Growth Factor Receptor; ALK: *ALK* Receptor Tyrosine Kinase; ROS-1: ROS proto-oncogene 1, receptor tyrosine kinase; PDL1: Programmed Death-Ligand 1.

**Table 2:** Pattern of Treatment: Radiation and Systemic Therapy.

Patients’ Characteristics	
PTV (cc) Median	392 cc (range 36.6–1600 cc)
Total Radiation Dose	
50 Gy	74 patients (35.2%)
55 Gy	102 patients (48.5%)
56 Gy	30 patients (14.3%)
60 Gy	4 patients (2%)
Chemotherapy	113 (53.8%)
Platinum-based chemotherapy	
+paclitaxel	2 patients (2%)
+gemcitabine	56 patients (49%)
+vinorelbine	12 patients (11%)
+pemetrexed	29 patients (26%)
Other in monotherapy	12 patients (10%)

**Table 3:** Treatment-related toxicities.

	**Grade 1**	**Grade 2**	**Grade 3**	**Grade 4**	**Grade 5**
Acute Adverse Events					
Asthenia	40 (19%)	6 (2.8%)	0 (0%)	0 (0%)	0 (0%)
Dyspnea	23 (11%)	2 (0.9%)	1 (0.4%)	0 (0%)	0 (0%)
Dysphagia	53 (25.2%)	34 (16.2%)	1 (0.4%)	0 (0%)	0 (0%)
Cough	60 (28.5%)	8 (3.8%)	0 (0%)	0 (0%)	0 (0%)
Chest Pain	8 (3.8%)	0 (0%)	1 (0.4%)	0 (0%)	0 (0%)
Pneumonitis	0 (0%)	0 (0%)	0 (0%)	2 (0.9%)	1 (0.4%)
Late Adverse Events					
	**Grade 1**	**Grade 2**	**Grade 3**	**Grade 4**	**Grade 5**
Asthenia	5 (2.3%)	1 (0.4%)	0 (0%)	0 (0%)	0 (0%)
Dyspnea	10 (4.7%)	6 (2.8%)	0 (0%)	0 (0%)	0 (0%)
Dysphagia	5 (2.4%)	1 (0.4%)	0 (0%)	0 (0%)	0 (0%)
Cough	13 (6.2%)	1 (0.4%)	0 (0%)	0 (0%)	0 (0%)
Chest Pain	1 (0.4%)	0 (0%)	0 (0%)	0 (0%)	0 (0%)
Pneumonitis	1 (0.4%)	8 (3.8%)	3 (1.4%)	9 (4.3%)	1 (0.4%)

## Data Availability

The data presented in this study is available on request from the corresponding author.
